# Use of kidney trajectory charts as an adjunct to chronic kidney disease guidelines- a qualitative study of general practitioners

**DOI:** 10.1371/journal.pone.0305605

**Published:** 2024-08-29

**Authors:** Michelle Guppy, Esther Joy Bowles, Paul Glasziou, Jenny Doust

**Affiliations:** 1 Institute for Evidence-Based Healthcare, Faculty of Health Sciences and Medicine, Bond University, Gold Coast, Australia; 2 School of Rural Medicine, University of New England, Armidale, Australia; 3 Australian Women and Girls Health Research (AWaGHR) Centre, School of Public Health, Faculty of Medicine, The University of Queensland, Queensland, Australia; Bolton Clarke Research Institute, AUSTRALIA

## Abstract

**Objectives:**

Chronic kidney disease (CKD) affects up to 11% of the population. General practice is at the forefront of the identification of patients with declining kidney function, and appropriate monitoring and management of patients with CKD. An individualized and patient-centred approach is currently recommended in guidelines, but would be enhanced by more detailed guidance on how this should be applied to different age groups, such as use of a kidney trajectory chart. We explored the opinion of general practitioners (GPs) about the potential utility of kidney trajectory charts.

**Methods:**

Qualitative study interviewing 27 Australian GPs about their management of chronic kidney disease. GPs were presented with charts that plotted percentiles of kidney function (eGFR) with age and discussed how they would use the charts manage to patients with declining kidney function. GPs’ opinion was sought as to how useful these charts might be in clinical practice.

**Results:**

Most GPs were positive about the use of kidney trajectory charts to assist them with recognition and management of declining kidney function in general practice: e.g, comments included a “*valuable tool*”, “*a bit of an eye opener”*,*” will help me explain to the patients”*, “*I’ll stick it on my wall*.*”*. GPs responded that the charts could help monitor patients, trigger early recognition of a younger patient at risk, and assist with older patients to determine when treatment may not be warranted. GPs also thought that charts could also be useful to motivate patients and help them monitor their own condition.

**Conclusions:**

Use of percentile charts in conjunction with the current CKD guidelines help support a patient-centred model of care. Kidney trajectory charts can help patients to understand their risk of further kidney damage or decline. Research on the use of these charts in clinical practice should be undertaken to further develop their use.

## Introduction

Chronic kidney disease (CKD) is a prevalent condition affecting between 2.5–11.2% of the adult populations of Australia, Europe, Asia and North America [1]. It is associated with common lifestyle diseases such as obesity, hypertension and diabetes [1]. General practice and primary care is at the forefront of the identification of patients with declining kidney function, and appropriate monitoring and management of patients with CKD [2]. Guidelines for the identification and management of CKD have been available for 20 years [3]. International guidelines have been recently updated, and feature special consideration for specific populations including older patients [4]. Different age groups are associated with differences in risks and long-term outcomes from renal impairment. For older patients the guidelines recommend a personalized approach that takes into account multimorbidity, frailty, cognitive function, polypharmacy and end-of-life care [4]. This patient-centred approach would be enhanced by more detailed guidance on how this should be applied to different age groups. It is not always clear how to apply shared-decision making with patients, as the outcome of a patient’s lower kidney function is not always apparent. For this reason GPs and primary care physicians need clearer guidance on the likely trajectory of a patient’s kidney function.

For younger patients, identification of those at risk can be more difficult. Younger patients with an estimated glomerular filtration rate (eGFR) above the abnormal range (≥60 mL/min/1.73m^2^) and without protein in the urine are not currently identified by the guidelines as being at risk. However, modestly reduced eGFR in young people is associated with adverse outcomes, including cardiovascular disease and death [5].

The current fixed threshold of an eGFR of 60 mL/min/1.73m^2^ to define chronic kidney disease may lead to missed diagnosis of kidney problems in younger patients, and overdiagnosis of declining kidney function in older patients [6]. Using an additional calculation that includes the percentiles of eGFR values with age, or means and standard deviations per age category, has been proposed as a method that might help prevent underdiagnosis and overdiagnosis of CKD [6]. A ‘kidney trajectory chart’ that plots eGFR percentiles with age could be a useful tool to show the normal distribution of kidney function with age, and its likely future trajectory. It could also help to monitor patients, allowing clinicians to re-evaluate the patient’s condition if they are dropping across percentiles [6, 7]. Our previous research showed that GPs using a kidney trajectory chart in two randomised case vignettes- an older patient and a younger patient- led to more appropriate recognition of the patients’ kidney function status and more appropriate management recommendations for both patients [8, 9]. In the present study we sought to determine GPs’ opinions of a kidney trajectory chart, and the acceptability of its use in clinical practice. We wanted to understand whether GPs thought this could be a useful tool and how it might be used as an adjunct to the kidney guidelines.

## Methods

This study was a nested qualitative component of a larger mixed-methods study. Participants were Australian GPs who had firstly participated in an online survey. GPs were initially recruited via email through an organisation called AMPCo. After participating in an online survey about CKD management, and a clinical vignette study, GPs could indicate whether they were willing to participate in a further interview about their management of CKD. GPs who responded were then purposively sampled and invited to do an hour-long interview about how they managed CKD in their clinical practice. Recruitment of GPs occurred between the 8^th^ August 2018 and 13^th^ November 2018, and the GPs were subsequently interviewed from May to June 2019. In the final component of the interview, the clinical vignettes and kidney trajectory charts that the GPs had originally seen in the online study were presented to them again. The GPs and the interviewer (MG- an academic GP) discussed these clinical cases and how they might apply the charts to their management decisions. GPs were asked a general question about the utility of the charts, and how they might use these charts on their real patients in clinical practice. The case vignettes, kidney trajectory charts and interview schedule can be seen in the supplementary files (S1 and S2 Files).

Ethics approval was obtained through the Bond University Human Research Ethics Committee. Prior to interview, GPs were sent a participant information statement with information about the research and interviewer. Written informed consent was obtained prior to the interviews taking place. Interviews were done using videoconferencing software, and were transcribed verbatim. Interview transcripts were emailed to the GPs for them to make any comments or corrections. Transcripts underwent thematic analysis by two researchers (MG and EJB) according to the method described in Braun and Clark [10]. Interviews continued until saturation of themes was reached. NVivo version 12 was used to assist in the coding and analysis process.

## Results

An email was sent to 9500 Australian GPs, and 469 responded. Of those, 399 completed an online survey, and 373 completed a clinical vignette study. Eighty-three GPs indicated that they would be available to be interviewed. Twenty-seven GPs were chosen purposively to be interviewed to ensure a balance of gender, age, and distribution of practice in Australia (urban, regional, rural, and spread across all Australian states and territories). There were no dropouts. Sixty percent of the GP respondents were female, with an age range from 31 to 70 years. Thirty-seven percent of respondents practiced in a remote location, 22% regional, and 44% urban.

### Reponses

Most of the GPs (89%, n = 24/27) responded positively to the kidney trajectory charts and said they would be useful in their clinical practice. Three GPs were neutral about them, and wanted more information on the chart to explain its use. None of the GPs were negative about the charts.

Most GPs made comments that the charts would be helpful: a “*valuable tool*” [GP2], “*it all makes sense… it would be a fantastic addition*” [GP38], “*it’s fairly straightforward*” [GP51], “*it’s quite user friendly*, *it’s quite easy to use*” [GP66]. With the three GPs who were neutral about the charts, they wanted some more information about using the chart: “*how to interpret the chart- that would be useful*” [GP19]. Another three GPs wanted to start using the charts in their clinical practice immediately “*I might even print it off and use it*. *Am I allowed to do that*?” [GP10] “*I think it would be quite useful to have this upper most in one’s mind or available as a resource*. *I’ll stick it on my wall*.*”* [GP27]

A common response to the chart was that it was a very useful tool for monitoring a patient over time, and giving some clear direction on indicating when further decline in kidney function was problematic.

“*I think it gives a bit more clarity into how to assess and monitor the kidney disease in the older age groups, that we were mentioning before in the guidelines it wasn’t necessarily, not that specific. That’s helpful. You could obviously plot that over time presumably for patients and it would be nice to have that even in your health record, because you could have a quick look at that and it would very quickly tell you where they were at and their rate of change*.”

[GP1]

“*As a GP*, *I would definitely like to chart every patient on this and would like to see where they fall into that percentile and this would give me a very good idea of we had to be more vigilant and more proactive in management of risk factors and medications*, *managing their lifestyle and prescribing ACE inhibitors and all that*, *controlling risk factors*.”

[GP35]

GPs also commented that the charts made it very clear when a younger patient was at higher risk, even if their eGFR was currently in the ‘normal’ range.

“*I think it helps very much with younger people…, but I think it’s extremely useful to highlight how abnormal a low eGFR is in a younger person.”*

[GP36]

“*I think it’s a bit of an eye opener to look*, *we sort of have this impression of GFR and what it should be and doesn’t look absolutely dreadful*, *but when you plot it all out on this chart*, *it’s quite sobering and you realise that relative to people of his own (age) … it’s either reassuring or concerning*. *It gives you a better perspective on what these people’s GFRs actually are*.”

[GP27]

Reflecting on their older patients, several GPs responded that it might alter their management in an older patient.

*“I think it would be useful to be maybe—to modify your approach when people are a lot older, yeah. So I think they’d be really good, they’d be super good for us, actually, because this is such bread and butter for us.*”

[GP10]

“*Having looked at the chart beforehand*, *look I would probably*, *in a 76-year-old*, *I’d probably treat them with a small amount of ACE*, *but looking at the kidney trajectory chart*, *possibly I wouldn’t bother*.”

[GP62]

However, one GP felt the chart wouldn’t change their management of older patients.

“*I’m not sure if it helps much with older people, just because I’m so hesitant to stop the things that they’re already on, or like I still feel the need to—I’m undecided, I’m ambivalent about starting things in older people*.”

[GP36]

GPs also described the chart as being a useful tool for patient motivation, or for patients to monitor their own condition.

“*I can show people something and I think if people see something, it means more.*”

[GP18]

“*Also it will help me explain to the patients as well that considering your kidney health*, *falls into this percentile*, *so it will also make them more involved in their care as well*.”

[GP35]

“*For the patients*, *you know*, *to try and spur people into taking action to control their own lives … you need something that means something to them*.*”*

[GP62]

There were a few components of the charts where GPs felt there was room for improvement. The first issue was around which population the chart was relevant for. Several GPs wondered whether this chart applied to Australian First Nations patients. Some GPs wanted further information about how to interpret the chart, such as a clinical decision aid. Two GPs commented on wanting further information on the chart, for example an indication of stages 3a and 3b CKD delineated on the chart. They also felt the age categories were a little hard to understand, and that either breaking into years of age, or smaller age gaps would be easier to determine. GPs gave varying views as to whether an electronic or paper format for the chart would be easier. A couple of GPs wanted to print the charts out and have them on their desk, or in a format they could give to patients. One GP described a paper-free office, and said the chart would be best integrated into their medical software, or on a website. Several GPs suggested that the chart should be integrated into pathology test reporting and automatically charted so that GPs could see the trajectory when reviewing patients’ pathology results.

## Discussion

### Summary of findings

The majority of GPs in our study were positive about the use of kidney trajectory charts to assist them with recognition of declining kidney function and management of CKD in general practice. Charts could help monitor patients, trigger early recognition of a patient at risk, and assist in determining when aggressive treatment may not be warranted in older patients. Charts could also be useful to motivate patients and help them monitor their own condition. Suggestions for different formats and charts for different ethnicities were discussed.

### Comparison with the literature

The concept of kidney age or using percentiles of function is not new, and has been proposed internationally [6, 11–13]. The normal rate of decline of kidney function with age has been regarded as 1 mL/min/1.73m^2^ per year, and occurs in healthy older people, not just those with disease risk factors [7]. The median eGFR in healthy younger adults is 106 mL/min/1.73m^2^, and begins to decline after age 40 [14]. Kidney function declines to <60 mL/min/1.73m^2^ in 40% older adults over 70 years [15]. Currently guidelines state that an eGFR of <60 mL/min/1.73m^2^ is common in older people but is nevertheless predictive of significantly increased risks of adverse clinical outcomes and should not be considered physiological or age-related [4]. Most older patients will never go on to have end stage kidney disease [16]. There are similar mortality risks for people with only mild reduction in kidney function (stage 3a CKD) and a normal urine ACR compared to those with normal kidney function [6, 16]. GFR performs similarly to other cardiovascular risk predictors, with urine ACR being the stronger risk predictor for cardiovascular mortality [17]. GPs in our study indicated that having a chart that made explicit this normal rate of decline of kidney function in visual form would be helpful for them to monitor their patients’ kidney function, be able to discuss risks with patients and make better decisions about when to monitor and when to intervene with their older patients. The trajectory charts presented here may form a useful adjunct to the well-validated Kidney Failure Risk Equation (KFRE) [18]. The KFRE equation predicts kidney failure risk in the subsequent 2 and 5 years, which can help to inform clinical decisions. Clinicians may, however, be concerned about an individual’s lifetime risk of advanced kidney disease, and comparing patients’ renal function trajectory with the age-matched general population is expected to assist in this assessment.

**Fig 1:**
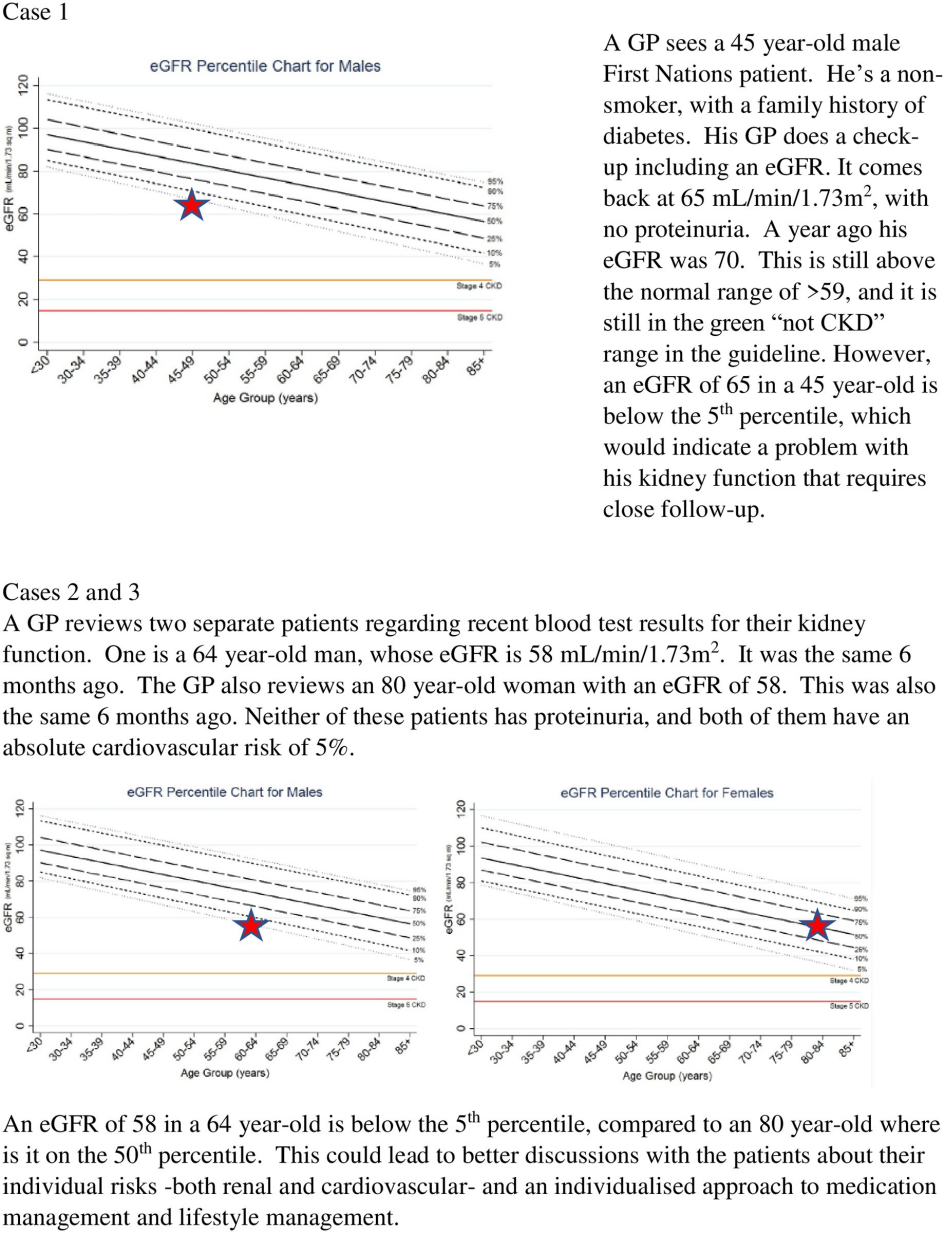
Example cases utilising kidney trajectory charts. GPs are very familiar with the concept of percentile charts, and regularly use paediatric growth charts, which are a similar concept. GPs already receive pathology data that they can track graphically over time, for example HbA1c in diabetic patients. Further research on the use of a kidney trajectory chart as an adjunct to the chronic kidney disease guidelines should be undertaken to see how GPs use these charts in the clinical environment. A trial of automated plotting of eGFR graphically by pathology companies would be a useful step to see how these might support the monitoring of patients with declining kidney function.

There is currently a gap in the guidelines relating to younger patients who might have a declining kidney function that is still above the reference range of 60 mL/min/1.73m^2^, but who are not identified as having CKD. A recent study found that 17% of young adults have a modestly reduced eGFR that is outside current guidelines for CKD threshold, putting them at risk for early cardiovascular disease and adverse outcomes [5]. Other recent studies highlight age related disparities for young people with moderate kidney function loss who aren’t classified appropriately in current guidelines [7]. If these young people are recognised early, their poor health outcomes may be prevented. As an adjunct to the guidelines, using percentiles of function according to age could aid in the appropriate classification of younger patients at risk [6, 7, 19]. GPs in our study indicated that the chart made a big difference in their ability to recognise risk in a younger patient with lower kidney function, and that it would be useful to have this as an adjunct to the guidelines to assist their assessment of risk. Fig 1 describes how these charts might be used in clinical practice.

### Limitations

One strength of this study is that it was conducted amongst GPs with a diversity of age, gender, and distribution of practice throughout Australia. The results of this study represent the views of the GPs who self- selected to participate in the interviews. This may not be representative of the broader GP community. However we continued to sample GPs until themes reached saturation in the interviews. Another limitation is the applicability to some groups of patients. Several GPs in our study questioned the relevance of the kidney trajectory charts to Australian First Nations patients and one GP asked whether they were relevant to Asian populations. The development of these charts has been previously described, and creatinine data was taken from the AusDiab study [8, 20]. The AusDiab study was a population based cross-sectional study of 11,249 community dwelling Australians conducted in 1999–2000 [20]. The Ausdiab sample underrepresented First Nations people, with 0.8% of the sample being First Nations, compared to ~2% of the Australian population [20]. The results of the Ausdiab study may not be generalisable to the First Nations or rural population of Australia, and may not be applicable to populations in other countries. Charts may need to be developed from different ethnic groups to reflect differences in kidney function between ethnic groups, for example the normal values of creatinine in a South-Asian population are different from the Western population [21]. However, there are also problems using averages from ethnic groups that may reflect socio-economic disadvantage rather than biological or genetic differences, and charts derived from these groups may perpetuate these disadvantages [22]. We only discussed two different patient clinical vignettes with GPs in this study. This may limit the responses of GPs, who may have considered the charts differently with a wider range of clinical scenarios. Further research needs to be done to test the charts with a broader range of clinical scenarios.

A few GPs described the need for more information on the charts to aid understanding. In our original study we deliberately did not indicate stage 3a and 3b CKD on the charts, as we were trying to determine GPs’ decision making without this information. We also did not provide detailed information on how to interpret the charts as we wanted to gain a clear understanding of how intuitive the charts were without a detailed decision aid. For use in clinical practice, charts would need to have clearer indications of how to interpret them to ensure consistency of use by clinicians. The charts do not include urinary albumin:creatinine ratio, which is an important part of the diagnostic criteria for CKD [4] and an important marker for cardiovascular risk [17]. Therefore these charts are not intended for use on their own, but as an adjunct to the clinical guidelines. There are inherent limitations in the estimation of eGFR, being based on serum creatinine levels, which need to be considered in any clinical decision making based on this measurement [23].

## Conclusion

Use of percentile charts in conjunction with the current CKD guidelines help support an individualized and patient-centred model of care. Kidney trajectory charts can help patients to understand their risk of further kidney decline or damage and should be done in conjunction with measurement of urinary albumin: creatinine, and an absolute cardiovascular risk approach. Further research on the use of these charts in everyday clinical practice should be undertaken to further refine their presentation and use.

## Supporting information

S1 File GP interview questions. (PDF)

S2 File Kidney trajectory charts. (PDF)
