# Supplementary material for: Use of kidney trajectory charts as an adjunct to chronic kidney disease guidelines- a qualitative study of general practitioners
Source: PLoS One. 2024 Aug 29;19(8):e0305605. doi: 10.1371/journal.pone.0305605 (PMC11361416; doi:10.1371/journal.pone.0305605)
Supplement: S1 File — (PDF) [file pone.0305605.s001.pdf]

Chronic kidney disease (CKD) management in older adults interview study- General Practitioner Interviews

PART D: CKD guidelines and older patients

1. How do you think the CKD guidelines relate to your patients who are 75 years or older?

Scenario 1

A 76 year old woman, whose only significant medical history is anxiety, is currently well and on no medications. She has no family history of heart disease or diabetes. She is a lifelong non-smoker and not diabetic.

She has an annual check- up with her GP. Her BMI is 27 and BP 140/90 (consistent with previous readings).

Her most recent results show:

Results	(Reference Range)
eGFR 58 (one year ago this was 60)	(>59 mL/min/1.73m2)
Urine ACR 2.0	(<3.5 mg/mmol)
Cholesterol 5.4	(3.9-5.5 mmol/L)
LDL 3.3	(0.0-4.0 mmol/L)
HDL 1.81	(1.1-1.9 mmol/L)
Fasting glucose 5.0	(3.6-6.0 mmol/L)

Please describe how you would manage this patient with respect to her kidney function.

Would your management change if she were 86 years old instead of 76 years old?

Then

Scenario 2

A 45 year old Aboriginal man lives in regional Australia with his wife and 2 children. He is an ex-smoker (he gave up 5 years ago). He is not diabetic, but has a strong family history of diabetes.

He has an annual check- up with his GP. His BMI is 27 and BP 140/90 (consistent with previous readings).

His most recent results show:

Results	(Reference Range)
eGFR 65 (one year ago this was 70)	(>59 mL/min/1.73m2)
Urine ACR 2.4	(<2.5 mg/mmol)
Cholesterol 5.4	(3.9-5.5 mmol/L)
LDL 3.3	(0.0-4.0 mmol/L)
HDL 1.81	(1.1-1.9 mmol/L)
Fasting glucose 5.0	(3.6-6.0 mmol/L)

Please describe how you would manage this patient with respect to his kidney function.

PART E: Kidney Trajectory Chart and CKD management

Provide GP with a copy of the "Kidney Trajectory Chart" for perusal

2. Please describe how you might use this chart in the management of the patient case/s above.

3. Do you have any comments about the Kidney Trajectory Chart and its relationship with the CKD guidelines in older patients?

4. Do you have any other final comments about the CKD guidelines or managing CKD risk in older patients?
