# Supplementary figures and images for: Use of kidney trajectory charts as an adjunct to chronic kidney disease guidelines- a qualitative study of general practitioners
Source: PLoS One. 2024 Aug 29;19(8):e0305605. doi: 10.1371/journal.pone.0305605 (PMC11361416; doi:10.1371/journal.pone.0305605)

## KIDNEY AGE TRAJECTORY CHART

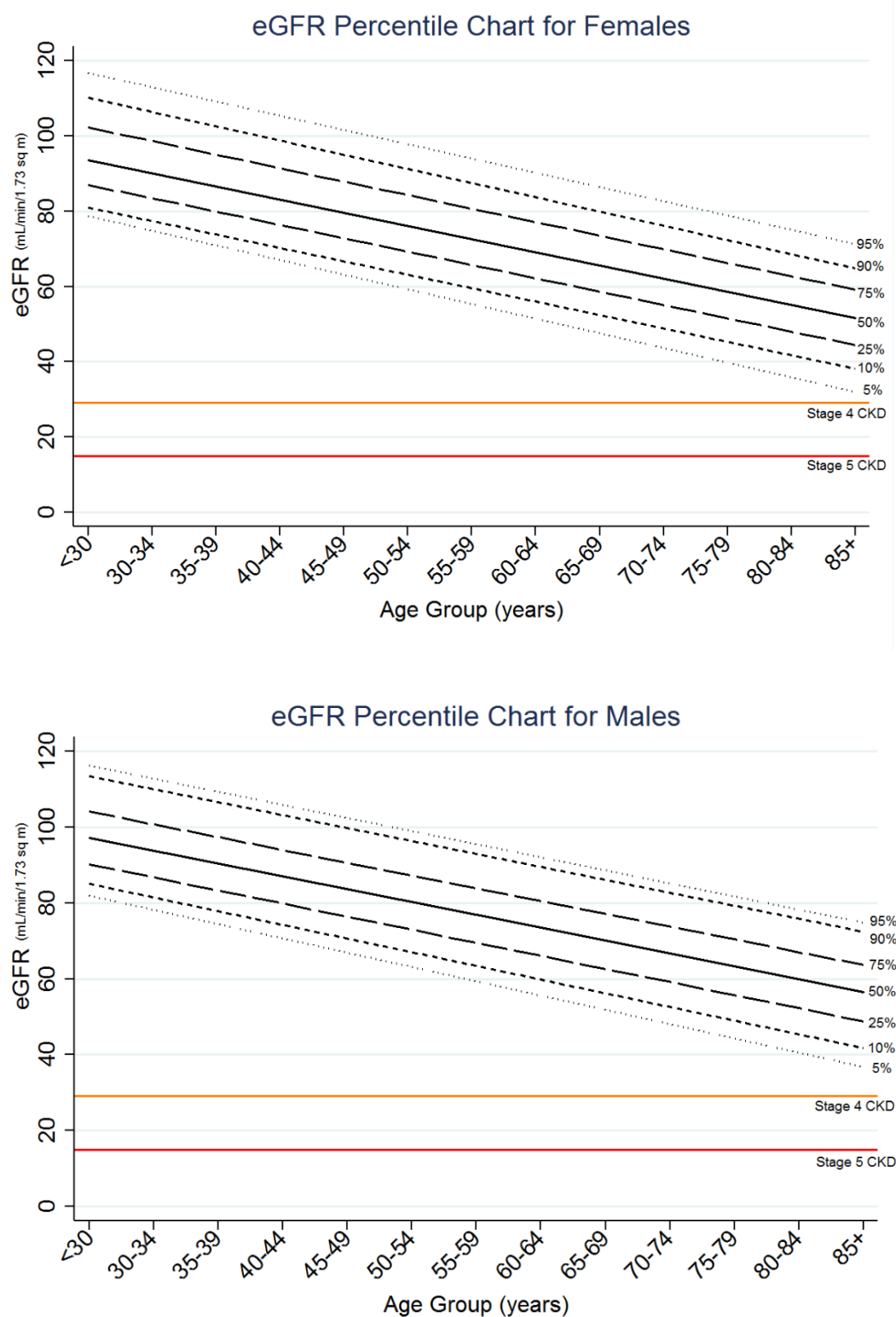

Supplement: S2 File — (PDF) [file pone.0305605.s002.pdf]
